# Creating an institutional ecosystem for cash transfer programmes in post-disaster settings: A case from Indonesia

**DOI:** 10.4102/jamba.v14i1.1046

**Published:** 2022-03-22

**Authors:** Jonatan A. Lassa, Gisela E. Nappoe, Susilo B. Sulistyo

**Affiliations:** 1 Northern Institute, Charles Darwin University, Darwin, Australia; 2 Institute of Resource Governance and Social Change, Kupang, Indonesia; 3 Social Protection Program, Wahana Visi Indonesia, Jakarta, Indonesia

**Keywords:** cash transfers, cash and voucher programming, institutional constraints, humanitarian ecosystem, post-disaster governance, Indonesia disaster management

## Abstract

Humanitarian and disaster management actors have increasingly adopted cash transfer as an approach to reduce the suffering and vulnerability of the survivors. Cash transfers have also been used as a key instrument in the current coronavirus disease 2019 (COVID-19) pandemic. This article uses an exploratory research strategy to understand how non-governmental organisations (NGOs) and governments implement humanitarian cash transfer in a post-disaster setting. This article asks: what are the institutional constraints and opportunities faced by humanitarian emergency responders in ensuring an effective humanitarian cash transfer, and how do humanitarian actors address such institutional constraints? In this article, we have introduced a new conceptual framework, namely the humanitarian and disaster management ecosystem for cash transfer. This framework allows non-governmental actors to restore complex relations amongst state, disaster survivors (citizen), local market economy and civil society. Mixed methods and multistage research strategies were used to collect and analyse primary and secondary data. The authors conclude that by implementing cash transfers in the context of post-tsunamigenic earthquakes and liquefaction hazards, NGOs must co-create an ecosystem of response that aims to restore disaster-affected people’s access to cash and basic needs. However, in order to ensure such access to basic needs, the responders must first restore relations between the states and their citizens before linking the at-risk communities with the private sectors to jump-start local livelihoods and market economy.

## Introduction

### Background

As an instrument for disaster and humanitarian response, cash transfer has gained global interest from the world humanitarian industry over the last 30 years. The recent global efforts to ensure that international aid reaches vulnerable and disaster-affected populations have been marked by the ‘Grand Bargain’ stipulated by the World Humanitarian Summit (WHS) 2016. The Grand Bargain sets out 51 commitments that are strategically organised into 10 thematic workstreams, including the imperatives to facilitate more support and funding tools to local and national responders as directly as possible and increase the use and coordination of cash-based programming (United Nations 2016).

Since then, international humanitarian players have committed to transform their mode assistance from traditional model of disaster response and operations that heavily focus on external mobilisation and procurement from outside disaster affected regions. Some organisations have made a bolder commitment to shift 50% of their assistance using cash-transfer programmes (CTPs) (World Vision 2019). In Indonesia, both humanitarian organisations and government have been collaborating with experimenting the use of CTP. For example, the Ministry of Social Affairs (MoSA) have been the focal point of the national CTP. The experiment of cash transfers by non-governmental organisations (NGOs) in collaboration with the government for disaster response in Indonesia has been implemented in Aceh after Indian Ocean Tsunamis.

In Indonesia, mainstream post-disaster and emergency CTPs from the governmental agencies are regulated by the MoSA Regulation No. 5/2015 (KEMENSOS 2015). The subjects of this regulation are both MoSA and local governments’ Department of Social Affairs (DoSA) at both district and provincial levels. The MoSA has been the cash transfer focal point in development context. It has been implementing a number of CTP-related programmes that aim at poverty alleviation and ensuring social development and protection in Indonesia since the last decade. The MoSA has recently been the leading agency for humanitarian cash transfer in Indonesia as it leads and coordinates both national and international humanitarian cash transfers through the humanitarian cluster systems. With or without the support of other ministries, MoSA, in coordination with DoSAs, often coordinate and facilitate local level arrangement of post-disaster related cash transfers.

The formal arrangement of cash assistance for disaster survivors is regulated by the MoSA. Unfortunately, apart from the coordination via humanitarian cluster mechanism in disaster response operation, there are no clear rules regarding how NGOs should implement their cash assistance. Therefore, most of the approaches are still informal and often implemented by NGOs in close coordination with local and national government agencies such as the MoSA and the DoSA.

The key objectives of disaster-related CTP under MoSA are: first, to ensure that the basic needs of survivors are met; second, to provide a well-targeted and efficient stimulant assistant for recovery and social protection; third, to ensure an accountable rehabilitation, recovery and relocation of survivors (KEMENSOS 2015). The MoSA’s CTP can be used for payment for building materials, living allowance, transitional housing, heirs, empowering the economy of disaster survivors, economic support for former combatants (in the context of post-conflict response) and support for villages where displaced and uprooted people are concentrated (KEMENSOS 2015).

International communities through Cash Working Group (CWG) – a mechanism under a sub-cluster Cash Transfers and Social Protection – distributed their CTP programme for at least US$ 25.2 million CWG in Central Sulawesi during November 2018 – December 2019. About 3.2 million out of the total were distributed in the form of cash assistance by MoSA in cooperation with DoSA, whilst the rest were distributed by NGOs.

### Cash transfer experiment in recent disasters and COVID-19

The tsunamigenic earthquakes that also triggered large-scale liquefactions in Central Sulawesi on 28 September 2018 had resulted in 2081 casualties, 1075 people missing about 211 000 displaced people and 68 000 damaged houses (BNPB 2018b). The total economic loss was estimated at USD 910 m (IDR 13.8 trillion) (BNPB 2018b) or about 350% of the total development budget of the Central Government Province in 2019 (Pemda Sulteng 2019b). One month prior to this event, a few big earthquakes shattered West Nusa Tenggara province, which claimed 436 lives and caused economic losses and damage of more than IDR 5.04 trillion (BNPB 2018b).

The government and humanitarian organisations (such as Wahana Visi, Catholic Relief Services [CRS] and Red Cross societies) responded to these series of disasters including two smaller events in Indonesia in the end of 2018 with the use of cash transfers. During COVID-19, the use of cash transfer has been escalated.

The Central Sulawesi earthquake and tsunami in September 2019 caused the isolation of most of the affected areas for several days. Electricity and telecommunications were cut off. Airports and ports, which were relied on for fuel distribution, were badly damaged. Landslides blocked several main roads. Disasters also impacted agricultural land, irrigation systems, fisheries, horticulture and markets. After the earthquake, many markets were closed and some were operating at only 50% of their capacity. Poor road conditions, damaged infrastructure and unavailability of transportation hindered access to markets in most of the affected areas.

### Background motivation and research questions

We were motivated to examine the complexity of institutional dimension, including formal institutions (government regulation, government organisations, laws, formal market, etc.) and social-informal institutions (non-governmental organisations, culture, values, tradition, informal market, religion, etc.) of a CTP in Indonesia. Wahana Visi Indonesia was selected as a case study in this research because of its significant contribution to the basket of cash assistance in Central Sulawesi (for about 10% of the overall interventions from national and international agencies).

It is essential to learn from experience using vouchers or cash transfers to meet people’s basic needs (Rutkowski 2018). Therefore, instead of showing another evidence of the power of cash transfers in humanitarian setting from other low- middle-income countries affected by another disaster, we use an explorative research strategy intending to understand how humanitarian cash transfer is implemented.

This article asks: what are institutional constraints and opportunities faced by humanitarian emergency responders, in ensuring an effective humanitarian cash transfer; and how humanitarian actors address such institutional constraints.

## Theories and concept of cash transfer for disaster and humanitarian response

### Brief overview of cash transfer as social protection

Mainstream academic literature often treats cash transfer as primarily a social protection strategy (Slater 2011). In the context of poverty reduction, cash transfer is cash assistance paid either by government or NGOs to poor households (Miller 2011). In the context of shocks from natural hazards such as droughts, cash transfer aims to reduce risks and vulnerabilities of the affected households (Devereux & Sabates-Wheeler 2008; Sabates-Wheeler & Devereux 2010).

Suppose development can be defined as an opportunity to expand human freedoms (Sen 1999). In that case, disasters and pandemic events, on the contrary, can be defined as a direct threat to development through compromising human freedoms and human insecurity. Furthermore, deprived freedoms and capabilities can lead to different forms of human insecurities, including food insecurity and hunger.

To what extent a person can cope with such crisis triggered by the events depends on the ‘entitlement basket’ ranging from producing food (production-based entitlement), buying food (trade-based entitlement), working for food (labour-based entitlement) and getting food aid (transfer-based entitlement). The potential impacts of disasters on the survivors’ access to basic needs can be explained by the classical food entitlement theory (Devereux et al. 2020; Sen 1983).

In many cases, the original objective of cash transfer is to enable low-income families to access food (Slater 2011) and non-food needs (Sphere Standard 2018) in the time of perils, such as disasters, including droughts and pandemics.

### A libertarian paternalism project

Conventional post-disaster aid distribution (in the form of commodity transfers, e.g., food and non-food items) is grounded in the moral imperative of paternalism, where external actors decide what is best for survivors of disasters and conflicts. On the contrary, cash assistance can be seen as a more flexible and relatively less-intrusive type of aid that is rooted in the ideology of libertarian paternalism (Tahler & Sunstein 2009) because peoples’ choices towards emergency aid are not ‘coercively enforced’ but creatively embedded in a new practice where people affected by disasters can experience a higher degree of agency and dignity (United Nations 2016).

To some degree, giving money to vulnerable people using cash transfers can be called a ‘real utopia’ (Bregman 2018). We argue that it is ‘real’ because mounting evidence of the positive impact of cash transfers in humanitarian emergencies can already be seen from many contexts since the last decade. Unconditional cash transfer (UCT) is a form of ‘basic’ income. Unfortunately, it remains a ‘utopia’ because, despite accumulated evidence of its effectiveness in both humanitarian and development contexts from around the world, policy makers and humanitarian actors remain reluctant about the use of cash transfer because of the perception of associated risk including security, inflations and anti-social use (Bailey, Savage & O’Callaghan 2008).

The increase in the use of cash transfer as a key instrument in fighting poverty and vulnerability can be seen as a silent revolution in international development studies and practices since the early 2000s (Gliszczynski & Leisering 2016). Nevertheless, the CTP took about a decade to emerge as a new strategic disaster response instrument. The WHS 2016 created a global momentum for cash programming as the summit secured a grand deal for ‘cash-based programming and more direct funding to local actors as critical operational measures for increasing efficiency, supporting people’s agency and stimulating local economies’ (United Nations 2016:14/22).

### Humanitarian and disaster cash transfer programme

Humanitarian CTP has gained popularity in the last decade (Doocy, Tappis & Lyles 2016) and it has become more common today and is known as ‘one of the most significant areas of innovation in humanitarian assistance’ (UNOCHA 2020:76). Such innovation has been a widely adopted practice in international development context (Leisering 2019). The merits of CTP have been well recognised in development studies literature and recently in disaster and humanitarian studies literature. For most of the proponents, there is little disagreement on the merits of CTP in improving humanitarian outcomes (Egeland 2018).

The CTP for both humanitarian and disaster response is more cost-effective as it has much lower transaction cost compared with conventional commodity aid relief that demands big logistical management costs. Cash has high convertibility in exchange; it also allows for greater freedom for the recipients and can stimulate local markets (Peppiatt, Mitchell & Holzmann 2001; United Nations 2016:14/22).

Cash brings some degree of freedom that can support the survivors of disasters to ‘prioritise their needs and meet them in a dignified way, which helps to stimulate markets and speed up recovery’ (UNOCHA 2020). The current ‘statement from the principals’ of four big United Nations agencies on cash assistance in December 2018 boldly underlines international commitment to promote cash-based assistance with suggestion for improvement in joint data management systems to avoid duplication of efforts (OCHA et al. 2018).

Remarkably, where markets were disrupted by war and conflict, CTP remain as one of the most favourable form of supports in almost all sector by household participants in Syria (Doocy et al. 2016). Cash transfer programme though has been widely accepted in remote communities in the Pacific, it remains underutilised because of lack of experiments from the existing humanitarian actors (Hobbs & Jackson 2016). And notwithstanding pessimistic view of the current world humanitarian system as a broken system, critics have been interestingly positive about cash-based interventions (CBIs) including cash and voucher programming (CVP – hereinafter, CTP will be used interchangeably with CVP and CBIs) as a form of innovation where partnerships with the private sector is recommended (Overseas Development Institute 2015; Spiegel 2017). Cash-based interventions/CVPs are also seen as a form of social protection that not only help disaster and conflict survivors to meet their basic needs (Thompson 2014) but also achieve humanitarian outcomes whilst helping local markets to recover (United Nations 2016).

Meanwhile, humanitarian and development actors have been thinking of the possibility of integrating CTP with social protection policy to address social-economic vulnerabilities (Gentilini, Laughton & O’Brien 2018).

Some of the concerns around cash transfers are central to the question of how effectively (local) markets will be able to respond to an injection of cash (Harvey 2007). Other concerns with the question: ‘will people be able to buy what they need at reasonable prices?’ or how can markets that may be particularly constrained or disrupted in conflicts and during disasters be functioned in developing countries (that are often weak and poorly integrated)?

Whilst many scholars agree that cash transfers can bring material benefits, some have also identified some potential drawbacks of using cash transfers. For example, experience from Zimbabwe suggested that resentment and tension might arise from targeting the beneficiaries (MacAuslan & Riemenschneider 2011). Even when the targeting is right, community perceptions might differ as the recipients might be seen as the winners whilst the non-recipients might be seen as losers. As a result, despite the potential impacts on material well-being, CTP could potentially have non-material impacts, for example, relational issues between the recipients and the non-recipients, including compromising social cohesion and social capital (Pavanello et al. 2016).

Other potential drawback of humanitarian cash transfer includes the potential to undermine the importance of informal risk-sharing arrangements (such as reciprocal lending and borrowing and other traditional forms of informal social protection, including social cohesion and social capital). Context also matters. For example, in the case of post-war or post-civil war, high convertibility of cash might allow certain groups to easily access illegal weapons to prolong violence (Willibald 2006).

One of the most significant challenges in implementing CBIs in vulnerable and fragile worlds is the lack of a regulated cash transfer system. For example, the bulk of humanitarian money in Syria is currently transferred through informal value transfer networks (hawala), which appears to have the capacity to handle larger-scale CTP (Doocy et al. 2016). As an evidence of multiplier effects of cash in both the humanitarian camp economies (of both refugees and internally displaced population), CTP/CBIs have been reportedly positive in many contexts (Abu-Hamad, Jones & Pereznieto 2014; Doocy et al. 2016; UNOCHA 2020). A comparative study from Congolese refugee camps in Rwanda suggest that the camps with cash aid appears to increase refugee welfare whilst strengthening market linkages between camp and host economies (Alloush et al. 2017).

The use of cash transfers in humanitarian settings has been relatively new (Abu-Hamad et al. 2014). Peer-reviewed publication, despite growing, remains limited. Most of the publications were made available by global think tanks such as Overseas Development Institute in London. Interestingly, most publications regarding the cash transfer focuses on showing evidence of its effectiveness in disaster response.

There is still a lack of understanding on the institutional dimension of cash transfers including its constraints and opportunities. Some research identified both hypothetical and empirical constraints. For example, Doocy et al. (2016) identified lack of clarity of institutional mechanism including ‘regulated cash transfer system for movement of funds into the country’ in the context of Syria (Doocy et al. 2016:2). Doocy et al. also highlighted that the feasibility of CBIs/CBPs relies on local context, which requires understanding of local ‘capacity, resources, political environment, beneficiary needs and preferences and lessons learned from previous programmes in those areas’ (Doocy et al. 2016:10). Unfortunately, the local institutional context is not adequately identified whilst others such as Abu-Hamad et al. (2014), Alloush et al. (2017) and MacAuslan and Riemenschneider (2011) were reluctant to use institutional settings.

This article is a proponent of cash transfer programmes for disaster and crisis settings including pandemics such as COVID-19. Such program can offer a more cost-effective disaster response system where humanitarian organisations and disaster managers can mobilise more resources directly to local communities including other local actors such as market and civil society. Cash transfer can also facilitate critical operational measures for increasing efficiency and better humanitarian outcomes (United Nations 2016).

The article addresses two research gaps. First, there is a lack of peer-reviewed literature on cash transfers from Southeast Asia and Indonesia – one of the world’s most disaster-prone regions. Second, this article focuses on the institutional dimension (see section ‘Research design and methods’) of cash transfer implementation that is often overlooked by previous publications.

Obviously, implementing CTP is not a simple process as many variables are controlled by others outside the command structure of any implementing agency. Unfortunately, these documented learnings do not provide an adequate account of institutional issues and real constraints faced by emergency responders and how such responders navigate through the institutional landscape to achieve an effective humanitarian response.

Despite a growing grey literature on humanitarian cash transfers at the international level, there remain limited documented empirical cases from Southeast Asia, including Indonesia. Furthermore, unlike CTP in poverty and development studies and practices, our observation also suggests that peer-reviewed literature on humanitarian/disaster-related CTP at global level is even less available.

### Operational framework

Humanitarian and disaster-related CTP is defined here as all type of programmes or interventions where cash or vouchers (for goods or services) are directly provided to individuals, household or community recipients (not to governments or other state actors) affected by disasters to enable them to meet their basic needs (World Vision 2017).

Cash transfer programme often uses conventional planning cycle: plan, operationalise, assess and control. For example, it should be assessed and delivered at the right time, at the right target group. With the right planning, humanitarian cash transfer can also complement and trigger local economy including local financial services (Taetzsch 2018). Therefore, the proponent of this approach must be able to create monitoring and evaluation measures (United Nations 2016) where the risks such as sudden inflation (Peppiatt et al. 2001) can be minimised. This requires a systematic monitoring and evaluation.

**FIGURE 1:**
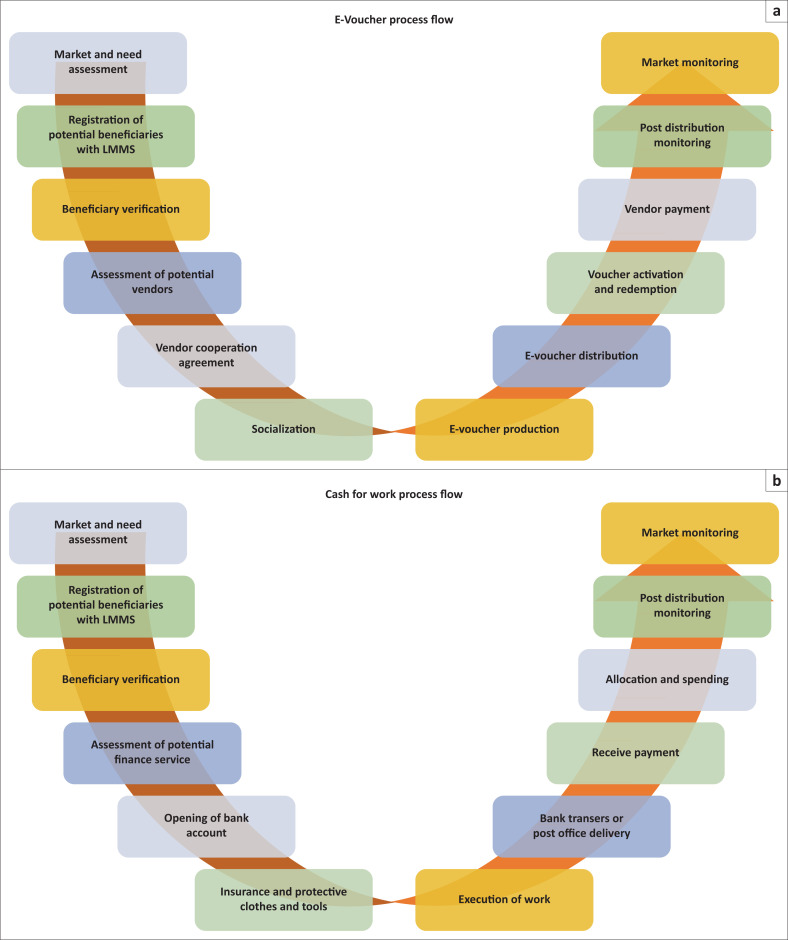
Illustration of work flows of cash for work and voucher.

The decision to select the best form of transfers (e.g. cash, vouchers, in-kind or a combination of all) must be based on a solid assessment including proper context analysis and the key important variables such as:

[*B*]eneficiary preference; cost efficiency and effectiveness; impact on the consumers and on the markets, including issues around market access; the availability of goods and services; risks associated with the transfer mechanism; as well as the overall impact of the project on the lives of children and vulnerable group. (World Vision 2017) (see also Figure 1)

Whilst acknowledging that cash is not a silver bullet, NGOs maintained the view that when partnering with governments and private sectors cash transfers can potentially facilitate social protection or safety nets including child sensitive social protection (Taetzsch 2018). It also envisions the use of cash or voucher as a tool to connect the consumers with the suppliers (World Vision 2018).

**FIGURE 2:**
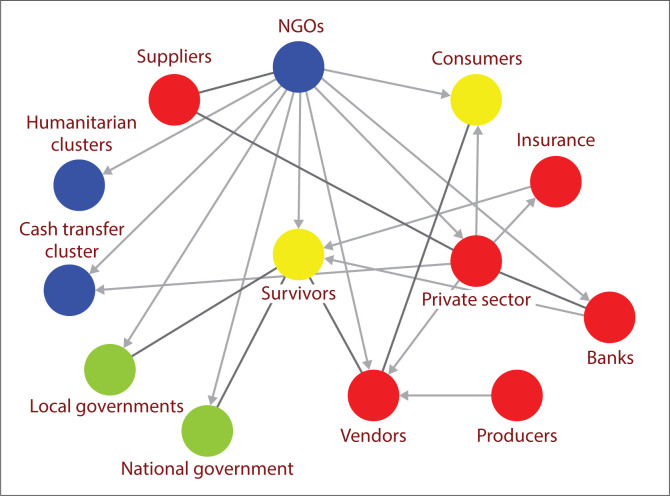
Illustration of an ecosystem of post-disaster cash transfer.

In order to perform an efficient and effective CTP programme, NGOs need to be mindful about local landscape of humanitarian and disaster management ecosystems where they have been playing intermediary roles in terms of financial and non-financial brokerage (DFAT 2018; Lassa 2018). This suggests that humanitarian responders should be able to broadly frame their response within an ecosystem to help them to navigate their response (DFAT 2018). Figures 1 and 2 imply an assumption that in order to design cash assistance, humanitarian actors need to understand the local economy and market dynamics in the aftermath of disasters.

Therefore, implementing a successful cash transfer programming also depends on responders’ ability to play orchestral roles and to connect with local humanitarian and disaster management ecosystems because their performance depends on the achievement of others: affected communities; local governments (from village to district to higher levels); private sectors (vendors, banks, insurance and situation of market’s supply and demand); humanitarian clusters; NGOs (see Figure 2). We argue that it takes ‘whole stakeholders’ or ecosystem approach to ensure humanitarian CVP works for the affected population.

Nevertheless, we argue that NGOs’ approach to CVP/CTP can be understood from the paradigm of humanitarian ecosystem approach (see Figure 2) and post-disaster governance theory. Humanitarian ecosystem comprises network of organisms (actors and institutions) (DFAT 2018) that interact in an ecosystem ranging from local to national and international levels and across state and non-state players (Trias, Lassa & Surjan 2019).

Humanitarian ecosystems approach allows observers and responders to view all stakeholders as part of a system of networks that needs to be (re)connected, where in an ideal world each component interacts with other components. Every organism or organisation in that ecosystem has a role to play in addressing humanitarian needs and reducing vulnerability of disaster survivors (Trias et al. 2019). Government organisations and institutions are equally important as other stakeholders including civil society, sub-national governments, the private sector (banks, vendors and insurance), donors and other international humanitarian communities. Ultimately, grassroots organisations and communities affected by disasters are integral part of the ecosystem. We argue that humanitarian ecosystem approach allows better understanding on how localisation of humanitarian emergency response should look like.

It is important to note that Wahana Visi’s approach (see Figure 1) is not entirely unique to humanitarian response context in Indonesia. Some players such as Oxfam and CRS have also been playing an important role in humanitarian cash transfers and have become core participants of cash transfers working group. Whilst using similar approach to Wahana Visi (see Figure 1), the partners of Disaster Emergency Committee (DEC) (such as Oxfam, UK) and Swiss Solidarity have been using banks and vendors as key stakeholder in cash transfer. Negotiation with the banks could be frustrated and time consuming and delay the response as experienced by DEC’s partners (Lawry-White, Langdon & Hanik 2019). Whilst for CRS, instead of using banks to deliver the cash to targeted communities, CRS, for example, has been using slightly different approach as they engaged Indonesian Post Office for cash delivery service (Ramadhan, personal communication, Respondent 13, Table 1).

**TABLE 1:** Research instruments.

Research instrument	Population	Total participants	Remarks
1. Household survey	A total of 510 household interviews in post-distribution monitoring	A total of 510 respondents (239 male; 271 female)	July–September 2019
2. Focus group discussion (s)	Two FGDs	Six participants	November 2019
3. Participant observation	Three cash and voucher distributions	Four villages in two provinces	November 2019
4. Semi-structure interviews with key informants	Five local governments Eight vendors Two bankers Two insurance representatives Eight NGOs staff	A total of 25 respondents	November 2019
5. Desk review	Project evaluation documents	Four evaluation documents	Produced from July 2019 to January 2020

Post-disaster governance theory recognises the fact that after large-scale disasters, with the influx of national and international players, a more complex arenas or centres of authority emerge to manage the impacts of the disasters. The nature of such complexity is known as polycentricity nature of post-disaster governance (Lassa 2015), where responsibilities and authorities are distributed and shared by many diverse actors ranging from local emergency bureaucracies, social services, NGOs and business to national government and non-governmental agencies to international organisations including the humanitarian cluster communities and funders. Being mindful of such polycentric governance landscape is important. The polycentric nature of CTP suggests that the nature of decision making in humanitarian emergencies is distributed in many domains and across scales and levels (Lassa 2015; Trias et al. 2019).

## Research design and methods

### Data collection

In this exploratory research, we applied mixed methods using multistage research strategy where the data collection methods started with household survey during July–November 2019. The quantitative data were derived from Wahana Visi’s post-disaster monitoring (PDM) survey data sets collected in July 2019 by recruited 13 enumerators who were trained by Wahana Visi’s Monitoring, Evaluation and Learning Team to conduct household survey using structured interviews with 510 respondents (239 male; 271 female) during July–September 2019. This strategy helped to understand constraints and opportunities faced by local communities in accessing cash transfers.

Qualitative data were collected by PDM’s Focus Group Discussions [FGDs] during May 2019, followed by field works including participant observations and in-depth interviews during 04–13 November 2019 in Central Sulawesi and 17–22 November in Lombok. We also used institutional ethnography methodology as a strategy to understand the inner workings of cash transfers in real-time settings. This institutional ethnography was possible because Sulistyo has been involved both as a CTP specialist and the coordinator of Wahana Visi humanitarian cash transfer and the coordinator of CWG in Central Sulawesi since the project started.

The researchers (external evaluators) (Lassa and Nappoe) also conducted semi-structured interviews with 51 informants including cash and voucher 28 beneficiaries (households) and local stakeholders ranging from bankers, insurance, traders, NGOs staff and local governments during 05–25 November in Central Sulawesi and West Nusa Tenggara provinces (Table 1). We (first and second authors) also conducted two FGDs with Wahana Visi’s field staff to gather initial insights of the stories including the dynamics of the programme implementation including their challenges and opportunities. We also used participant observation strategy as we attended six cash and voucher distributions including one community meeting for cash and voucher distribution planning.

### Data analysis

The authors used qualitative data information collected from Table 1. We performed qualitative data analysis from various sources from Table 1 using Excel to tabulate the qualitative data from the survey and the qualitative data collection from interview. Coding table was used in the Excel file.

### Limitation

We did not have the time to compare the response from both transfer recipients (the intervention group) compared with non-beneficiary households (control group). The household survey was based on random selection of the beneficiaries, whilst the eligibility criteria for inclusion of beneficiaries in the semi-structure interviews were initially based on random selection from the list of beneficiaries. However, after the first day trial, we shifted to stratified random selection as we decided to prioritise the profile of most vulnerable group with criteria, namely families with children and children under five. The main reason for this is because the humanitarian cash programme had been designed to address children’s vulnerability.

## Findings

This section presents the empirical findings based on the exploration of how humanitarian cash transfer is implemented in the real-world setting. Key challenges and prospects for improvement of cash transfer programming in Indonesia are highlighted.

### Context of intervention

Following the disaster in Central Sulawesi, the National Disaster Management Agency (BNPB) received IDR 43.2 billion (USD 3.2 m) from international donors and distributed it as cash transfer through MoSA (Hikmat 2019). Furthermore about IDR 300 bn (USD 22 m) were allocated by NGOs from CWG in Central Sulawesi as of 2019. This brings a total of IDR 343.2 bn (USD 25.2 m) international cash assistance being distributed in Central Sulawesi during October 2018–November 2019.

This suggests that internationally sponsored CTP equals 18% of the total direct procurement from the Central Sulawesi Government budget during the fiscal year 2019 (about IDR 2 trillion) or almost 9% of the total entire Central Sulawesi Government’s budget for the fiscal year 2019 (Pemda Sulteng 2019).

Furthermore, using domestic grants, the MoSA/DoSA also allocated for the 1883 verified heir compensation (santunan alih waris or SAW). based on 1883 verified claims (Jayadin, Respondent 12 – Table 2). Thus, the total allocated payment was about Rp 20 bn, where each beneficiary received a total of IDR 15 m (USD 1100) (Hikmat 2019).

**TABLE 2:** List of key informants from semi-structured interviews.

Respondent IDs	Method	Position	Agency	Date
R01	FGD 1	Cash Officer and National Livelihood Specialist; Cash Field Officers	Wahana Visi	03 November 2019
R02	Personal interview	Cash Officer	Wahana Visi	04 November 2019
R03	Personal interview	Secretary	Central Sulawesi Civil Registration Office	13 November
R04	Personal interview	Owner	Sinar Belawa	08 November 2019
R05	Personal interview	Head of General and Logistics Division	Bank Sulteng (Central Sulawesi)	13 November 2019
R06	Personal interview	Relationship Manager	Bank BRI Lombok	21 November 2019
R07	Personal interview	Head of Branch	BPJS Labour, Donggala	04 November 2019
R08	Personal interview	Account Representative Officer	BPJS Insurance	21 November 2019
R09	Personal interview	Owner	Arini Jaya	08 November 2019
R10	FGD 2	Owners	Joint-venture	10 November 2019
R11	FGD 3	Owners	Joint venture	10 November 2019
R12	Personal interview	Head of Disaster Management Division	Department of Social Affair Central Sulawesi	07 November 2019
R13	Personal interview	Emergency Coordinator	Catholic Relief Services	09 November 2019

FGD, Focus group discussion.

The other type of CTP was a living allowance known as Jadup (funded by international assistance). As of 31 October 2019, at least 87% of the total fund has been distributed to about 16 000 households (64 000 headcounts). The Jadup is a form of cash transfer/social protection – where each disaster-affected individual members in a household received IDR10 000 per day for up to 60 days (or IDR 600 000 for two-month allocations) (KEMENSOS 2015). So, for example, if there is a five-member family, the household will receive IDR 3 m or about USD 440 for two months. Local DoSAs are the main counterparts for cash transfer in social development and humanitarian emergency context. In brief, DoSA distributes MoSA’s CTPs.

### Rules of the game of cash transfers

The inclusion of the Wahana Visi Indonesia’s (WVI) CVP participants (beneficiaries) was based on three inclusion criteria: (1) non-government staff, (2) not currently working with private sector and having salary above minimum wage; (3) affected by recent disasters and can be verified by village/community level officials (Respondent 01 and 02). The administration system of the CVP distribution requires every beneficiary to have valid personal identification documents (IDs). The IDs were also used to open bank accounts and insurance account. If IDs were lost in disasters, the replacement should be immediately provided.

Wahana Visi has implemented three main types of CVP in Central Sulawesi: cash for work (CFW), multipurpose cash assistance (MPCA) and vouchers.

The CFW is a conditional cash transfer where affected communities receive payment in exchange for labour allocated for recovery activities, including community development activities. It has been implemented at different stages, from emergency to recovery stages, during 2018–2020. The first CFW was implemented in early 2019. The payment was based on the regional minimum wage, where in Central Sulawesi, each selected participant was paid IDR 80 000/person/day (each day is 6-h work). The actual payment was based on the actual number of work day.

The second type of cash transfer is MPCA transferred via banks. The MPCA is a type of unconditional CTP that beneficiaries can use to buy whatever they want. The size of the MPCA depends on the scale of the loss and damage of the house of the survivors. Three categories are applied to measure the damage: light damage, moderate damage and heavy damage. For light, moderate and heavy damage to the house, each household were granted IDR 1 m, 1.5 m and 2 m per month, respectively after the disaster in Central Sulawesi. Based on the consensus amongst the CGW and local governments, the maximum UCT (such as MPCA) was for three months (Pemda Sulteng 2019). In addition, governments also have UCT distribution (see next Section) with a maximum of two-month allocation. However, the allocation of UCT is constrained by the context of government and NGOs trying to avoid overlap. Furthermore, funding is uncertain as most NGOs often make emergency appeals for a shorter period.

The third type of CTP is vouchers which provide predefined goods and services based on community consultations and can be exchanged in specifically organised fairs or at designated service providers (World Vision 2017). For example, communities needed to have a kitchen set and household items during the early stage of the emergency phase in the disaster hit Central Sulawesi. Therefore, a voucher for a kitchen set with a value of IDR 1 m (USD 74) was distributed to each beneficiary household. In addition, each child from selected schools was given a school uniform voucher for IDR 100 000 (USD 7). Finally, we directly observed distribution of one agricultural voucher (including seeds and other agricultural inputs) in Central Sulawesi, where each household received IDR 3.13 m (USD 230).

### Roles of NGOs as connectors: Restoring citizen–state relations

This finding is a pleasant surprise, and it is a result of our explorative approach. Tsunamis and liquefaction and the earthquakes on 28 September 2018 damaged and destroyed thousands of houses, causing losses of lives and valuable assets, including valuable documents such as land certificates, house certificates and IDs. During our interviews, one of the traders shared his experience that he was not allowed to get milk and water from a distribution point controlled by a group of military members near his damaged shop. One reason was that he could not show his IDs as they were lost because of liquefaction that hit his house (Correspondent 04 – Table 2).

The moral of the given story is not about the good or bad of the involvement in untrained armies in humanitarian aid distribution. But instead, this shows a piece of evidence that disaster can cause loss of connection between a citizen (disaster survivors) and the state (governmental agencies) as disasters destroyed the ‘sacred papers’ (the IDs), consequently state–citizen relation is at risk. Therefore, disasters can potentially create new forms of social exclusion sponsored by the state as government officials might fail to establish their operation based on humanitarian principles.

Humanitarian NGOs often establish their beneficiaries’ registry before their aid distribution. At Wahana Visi, such a system is called Last Mile Mobile Solutions (LMMS) – a digital platform for beneficiaries’ registry (World Vision 2019). The LMMS outlines operational questions: What are the needs, who are the beneficiaries and how to identify them? What are the community structures? How to verify the identity of the beneficiaries? Which agency holds power to verify the demographic information of an affected population?

We observed that, in general, it was necessary for each beneficiary to show their formal IDs, without which they were not included in such a registration system whether being implemented by NGOs or local governments (e.g. DoSA). Whilst we asked 28 interviewees (beneficiaries) during 03–24 November 2019 regarding their barriers to access CVP supports from Wahana Visi, 7 out of 28 interviewees said they lost their IDs but mostly were able to get their IDs in time because NGOs facilitated the process. Only 4 interviewees mentioned their concern about the cost to reclaim their IDS because of distance and transportation cost.

Our findings from Central Sulawesi are well-founded because, in times of emergencies, NGOs must work quickly to collaborate with the local governments from village to district/provincial levels to speed up the process of IDs replacements. Therefore, despite not being seen directly as important and necessary by many specialists and non-specialists, government/local governments – NGO partnerships in post-disaster civil registration services are vital.

Civil Registration Agencies (CRA or Disdukcapil) have been identified as one of the least known agencies in the aftermath of disasters (Respondent 03) – indicated by their absence in most local disaster regulation (e.g. see Gubernur Sulawesi Tengah 2013). Yet, Disdukcapil/CRA has critical roles in disaster response, as recently demonstrated in Central Sulawesi tsunamigenic earthquakes. Two weeks after the disasters, there was a mounting demand from local and national response agencies to validate and verify their data. Disdukcapil found that their role was critical in ensuring better targeting and identifying the authenticity of victims and survivors (Respondent 03). Without their prompt actions, the quality of social protection and humanitarian services will be affected.

The first team of the Director-General of Civil Registration (DG Capil) under the Ministry of Interior (MoI) arrived in the capital of Central Sulawesi six days after the tsunami/earthquakes and liquefaction. The team brought 10 000 blank ID cards (namely e-KTP), including recoding and printing machines. Anticipating the tremendous demand for ID replacements to support anticipated big aid distribution from governments and NGOs, the Central Sulawesi governor and the DG Capil activated the civil registration services on 04 October 2019. The service was fully operational 24/7 since 04 October for the next four months (Dasmud, Respondent 3, Table 2).

As the role of such civil registration agencies is not well recognised by both mainstream actors including local disaster management agencies, the CRA had minimal resource (e.g. logistics such as accommodation) to operate post-disasters. Therefore, some of the incentives (including lunch/dinner) were provided by CWG/NGOs to support Disdukcapil to work in weekends during emergencies (Dasmud, Respondent 03). As a result, two channels were open for ID replacement services. First was the regular channel where any concerned citizen could request new IDs from Monday to Friday. The second channel was an emergency or NGO channel where Disdukcapil provided a speed up replacement process facilitated (including evidence for ID verification) by NGOs, including CWG members (Dasmud, Respondent 03). These processes were also observed and co-facilitated by the authors (third author).

### Private sector engagements

#### Exposure to banking

Data from the household survey suggest that about 72.5% of the respondents (370 out of 510) did not have bank account prior to the distribution of MPCA. Therefore, this is a dramatic social change. About 70% (359 out of 511) respondents also said that WVI facilitated the process to open a bank account for them. Banks often demand a rigid administration procedure including identification of account holders. As many lost their IDs during disasters, the NGO must work with local government to accelerate replacement of IDs to satisfy banks’ requirements. Only 6 out of 510 respondents said that the process was challenging but without citing any reason as to why, and only two respondents had problems with the disbursement of the money from the bank. One of these respondents mentioned unmatched signatures and other reasons such as unauthorised representatives. The disbursement process also varies, but most respondents (482 out of 510) went to the nearest branch to get their money. Whilst 13 respondents said that they used automatic teller machines, the rest mentioned that they got their money from agents and direct payment at the village office.

We were (first and second authors) able to participate in some of these processes during our fieldwork as beneficiaries and field cash officers come to the bank to process account opening. Likewise, we were able to observe how the recent shelter project from WVI facilitated Sulteng Bank to present terms and conditions for banking in a community meeting in Lero Tatari village in Donggala District. The survey data also confirmed that 92% (470 out of 510) of the respondents received information about such terms and conditions of banking.

Despite the temporary nature of the humanitarian cash transfers, where the beneficiaries often get cash transfers into their account for two to three months, the CVP activities have been exposed to the banking system as required by the rules set by the project. This was the case in both the Wahana Visi in Central Sulawesi. Interestingly, such a vision is also shared by MoSA (Hikmat 2019). But, unfortunately, we had no access to information regarding how many bank accounts remained active three months after distribution.

The motivation of banks’ participation in the humanitarian cash transfers varies, as they could be a mixture of social action, purely for business reasons, or political and economic reasons. Our interviewees stated that whilst there is only marginal benefit in the short run because of the nature of the humanitarian money that ‘flying in but flying out very quickly’ (Iqbal – Respondent 05 – Table 2), there is also the potential for the long-term benefit of marketing. Therefore, for the banks, the CVP certainly brought some degree of social service but participation in CVP can also promote banks’ interest in the long run (Respondent 06 – Table 2). What is important to note is that the participant banks were either state-owned or local government-owned enterprises. Therefore, their participation was to some degree mandatory as required by local/national governments. (Respondent 05, 06).

There were spikes of demand for banking services during the emergency stages, however the banks lacked requisite staff for field distribution and direct community engagements. For example, ‘thousands of beneficiaries who participated in CFW demanded that the bank should send staff/teller to the field. In addition, in post-disaster settings, cash distributors often pushed the bank staff to work overtime during weekdays and even the weekends’ (Respondent 05). However, in Central Sulawesi, the beneficiaries’ registration was not a significant hurdle for the banks because there was a rapid response from the NGOs and the local government after the 2018 tsunamis. Most local governments in the province from sub-village to district levels were able to operate a few weeks after the 2018 disasters.

#### Exposure to insurance

In Central Sulawesi, almost 95% of all the beneficiaries signed up for occupational hazard protection insurance (BPJS-K) for the first time when joining CFW project. This has been consistent across Central Sulawesi (Respondent 07). The BPJS-K insurance covers protection for work accidents and loss of life. The payments for the insurance were made via bank transfer with a premium of IDR 11.000 (USD 0.80). Each worker is entitled to a pair of safety shoes, a helmet and proper tools. Children are not allowed to work (Respondent 01 – See Table 2).

Like in Central Sulawesi, BPJS-K in Lombok (West Nusa Tenggara) said no observed independent continuation of the insurance after the project. However, the respondents stated that:

‘BPJS-K were fortunate to involve in WVI cash transfer because CFW can be used as an insurance and protection literacy project to educate people about workplace safety and while marketing for the future’. (Respondent 08)

### Can cash and voucher programming nudges local market economy recovery?

In general, despite being able to make progress in disaster risk management systems in the last 10 years, we observed that local governments in Indonesia will still need a more detailed plan to help the private sector recovery in the long run. Therefore, it is not an exaggeration to say that NGOs’ CVP can nudge the cash economy in one way or another. This view is supported by a vision from the MoSA where ‘financial literacy’ was cited as one of the side objectives of cash transfer (Hikmat 2019).

The household survey data in Central Sulawesi suggests that 71% of the household buy their food and non-food items from traditional markets. Only 3 out of 511 respondents cited shopping mall as their destination. The CVP, therefore, could speed up local traders’ recovery. In Central Sulawesi, most vendors were also survivors who lost either half to most of their strategic assets such as houses and shops. One type of voucher programme was kitchen sets. The vendors agreed to participate in a bazaar for kitchen sets. On average, signed contracts ranged from USD 7000 to 15 000. At least 7 out of 8 interviewed vendors who participated in the bazaars for voucher distributions cited that such an approach helps them to partially recover from disasters. One vendor who lost two of her private houses said that ‘this voucher deal helped us as we were able to stand on our feet again’ (Jumeha, Respondent 09 – Table 2).

Joining the voucher programme and participating in the bazaar also posed a business risk for the traders. However, for smaller vendors to participate in voucher programs, they tend to joint venture to enable them to bid for the commodities’ supply and distribution. One of the reasons for joint venture is to share risks. They cited that they had to take risk as they anticipate the benefit from the deal. For example, HM (Respondent 04), whose daily sales were only USD 500 per day, decided to procure orders with a value that is 20 times his daily sales. He argued that he had to take the risk to participate and cited that trust is a tricky business. One of the risk management measures for the vendor is triangulation of information about Wahana Visi from peers, consumers, media and internet, attending meeting/workshop and visit Wahana Visi offices and being deliberative in signing the contracts.

## Discussion

Most of the literature on humanitarian cash transfers, including CVA/CVP, primarily focuses on providing solid evidence on how cash transfer contributes to better humanitarian outcomes (Alloush et al. 2017; Gentilini et al. 2018). However, previous research suggested that cash transfers, including CVP-related activities, can only be successful if there is a functional market mechanism, including institutions (Harvey 2007; ODI 2015). The issue is what kind of institutional arrangements has been left unanswered because the institutional context and the nature of each crisis also matters.

However, institutional arrangements do not simply rest on the shoulders of NGOs and governments as more players have been involved in disaster settings. For example, unlike the traditional humanitarian players, CVA/CVP demands local traders and financial institutions that function as aid distributors.

Our findings suggest that the success of humanitarian cash transfers depends on a relatively complex institutional mechanism (Doocy et al. 2016). Ensuring access to the post-disaster civil registration system allows the survivors to access external assistance, including cash transfers from local governments, NGOs and access to protection (e.g. insurance) and banking services. Tsunamis, liquefaction and earthquakes cause losses of IDs and essential documents for thousands of survivors. However, losing a personal ID card in tsunamis is not a simple event. Disasters can immediately cut the ties between the state and their citizen. Without proof of identity, disaster survivors become stateless. States and aid registration system can potentially serve as social excluders (Asia Foundation 2016). The formal institutional system, including the banking system, needs to verify the survivor’s IDs before replacements can be facilitated. Unlike earlier understanding that social exclusion is the critical driver for socio-economic vulnerability and disaster creation, our findings suggest that disasters serve as excluders as they cut both social (between citizens) and civil ties (between citizen and the states). Therefore, we also argue that the first necessary response from NGOs in the post-disaster CTP is to ensure that the citizens reconnect with their state via fundamental civil rights, namely restoration of their IDs.

## Conclusion

Using explorative research, this article asks the questions: What are the constraints and opportunities in ensuring an effective humanitarian cash transfer? What are the barriers to (access) cash assistance in emergencies faced by the people, including the stakeholders affected by disasters? and How the humanitarian actors address the challenges.

We found that the first fundamental step to ensure cash transfer’ success is to restore access to civil registration services immediately after disasters. Losing IDs multiplies survivors’ vulnerabilities as they are potentially excluded by the state, the market and NGOs’ access to basic needs. Communities’ entitlements (to rights and protection services) are restored by nudging governments to recognise their citizen in perils, whilst at the same time nudging the markets to be channelled to meet humanitarian needs.

Unlike traditional aid distribution systems, cash transfers require a more complex arrangement at different levels, as visualised in Figures 1 and 2. We conclude that in trying to establish a mechanism for cash transfers to the affected community, the NGOs co-created an ecosystem required for cash transfers. In Palu, NGOs, including Wahana Visi, play roles as orchestrators of humanitarian response where NGOs connected the dots of the affected people (beneficiaries) with the governments and market. From this point of view, we can argue that NGOs participate in co-creating a disaster governance ecosystem in such a cash transfer project.

This research contributes a new understanding of disaster governance and CVP at three levels: Firstly, cash transfers and their success depend on the institutional settings after disasters. Secondly, NGOs and private sectors can play a significant role by co-shaping the local humanitarian ecosystem to protect the affected population. They can orchestrally co-govern the humanitarian ecosystem. Thirdly, the traditional understanding of governance suggests that governments can monopolise the steering privileges over the other actors in both disaster and non-disaster contexts (Lassa 2011). Findings from Central Sulawesi suggests that NGOs can transform the humanitarian landscape by their ability to condition and connect their peers, local government and private sectors to deliver cash services to the survivors.
